# Survey on the knowledge and practices in anorexia of aging diagnosis and management in Japan

**DOI:** 10.1002/jcsm.13566

**Published:** 2024-08-21

**Authors:** Sahoko Takagi, Shosuke Satake, Ken Sugimoto, Masafumi Kuzuya, Masahiro Akishita, Hidenori Arai, Ivan Aprahamian, Andrew J. Coats, Tatiana Klompenhouwer, Stefan D. Anker, Hidetaka Wakabayashi

**Affiliations:** ^1^ Department of Nutrition Management, Hospital National Center for Geriatrics and Gerontology Obu Japan; ^2^ Department of Geriatric Medicine, Hospital National Center for Geriatrics and Gerontology Obu Japan; ^3^ Department of Frailty Research, Research Institute, Center for Gerontology and Social Science National Center for Geriatrics and Gerontology Obu Japan; ^4^ Department of General and Geriatric Medicine Kawasaki Medical University Okayama Japan; ^5^ Meitetsu Hospital Nagoya Japan; ^6^ Tokyo Metropolitan Institute for Geriatrics and Gerontology Tokyo Japan; ^7^ National Center for Geriatrics and Gerontology Obu Japan; ^8^ Division of Geriatrics, Department of Internal Medicine Jundiaí Medical School Jundiaí Brazil; ^9^ Heart Research Institute Sydney New South Wales Australia; ^10^ Society on Sarcopenia, Cachexia and Wasting Disorders Lausanne Switzerland; ^11^ Department of Cardiology (CVK) of German Heart Center Charité, Institute of Health Center for Regenerative Therapies (BCRT), German Centre for Cardiovascular Research (DZHK) partner site Berlin Charité Universitätsmedizin Berlin Germany; ^12^ Institute of Heart Diseases Wroclaw Medical University Wroclaw Poland; ^13^ Department of Rehabilitation Medicine Tokyo Women's Medical University Hospital Tokyo Japan

**Keywords:** Anorexia of aging, Continuing education, Geriatric anorexia, Healthcare professionals, Older patients

## Abstract

**Background:**

Anorexia of aging (AA) is a condition in older adults that includes loss of appetite and reduced food intake. There is a lack of detailed analysis of the potential influence of educational initiatives in addressing AA. This study aimed to clarify the current state of knowledge and practice regarding AA and its relationship with the availability of continuing education opportunities among Japanese healthcare professionals involved in treating older patients.

**Methods:**

The Japan Geriatrics Society and the Japanese Association on Sarcopenia and Frailty, in collaboration with the Society on Sarcopenia, Cachexia, and Wasting Disorders, conducted an online questionnaire survey on the knowledge and practices in AA detection and management. Questions were asked in the areas of demographics, screening, definition/diagnosis, treatment, referral, and awareness, with those who ‘participate’ in continuing education and professional development programmes in nutrition for their patients were classified as the ‘education group’ and those who ‘do not participate’ were classified as the ‘non‐education group’. The results for each question were compared.

**Results:**

The analysis included 870 participants (physicians, 48%; registered dietitians, 16%; rehabilitation therapists, 14%; pharmacists, 12%; nurses, 6%; and other professionals, 5%). The education group (45%) was more likely than the non‐education group (55%) to use the Mini‐Nutritional Assessment Short Form (MNA‐SF) to screen for AA (49% vs. 27%) and less likely not to use a validated tool (33% vs. 47%). More participants used evidence‐based tools and materials for AA care (38% vs. 12%), and fewer used their clinical judgement (23% vs. 35%) or were unaware of the tools and materials (9% vs. 23%). The proportion using a team of professionals experienced in AA care were 47% and 24% of the education and non‐education groups, respectively. By profession, few physicians used specific validated tools and resources for AA screening and treatment. More than half of the dietitians used the MNA‐SF regardless of training opportunity availability. Regarding professional availability and team use, differences in educational opportunities were particularly large among physicians.

**Conclusions:**

Participation in continuing education programmes on nutrition is associated with responsiveness to AA screening and treatment and the availability of a team of professionals, which may influence the quality of AA treatment. Nutrition education may support the confidence of healthcare professionals working with older adults in AA with complex clinical signs and encourage them to conduct evidence‐based practice.

## Introduction

Loss of appetite and reduced food intake in older adults are common symptoms affecting approximately 30–40% of hospitalized older adults, 10% of community‐dwelling older adults, and 10–30% of nursing home residents.^1^, ^2^, ^3^, ^4^, ^5^ The prevalence of this syndrome increases with age, raising concerns that they may be perceived as an inevitable consequence of aging and thus overlooked without appropriate intervention despite having numerous reversible aetiologies.^4^, ^6^, ^7^

Untreated conditions increase the risk of malnutrition, sarcopenia, frailty, and cachexia, often culminating in adverse outcomes such as hospitalization and mortality.^2^, ^4^, ^5^, ^7^, ^8^, ^9^ Consequently, these symptoms in older people were conceptualized in the late 1980s as a geriatric syndrome termed anorexia of aging (AA).^10^, ^11^ Addressing AA has become imperative, particularly in aged societies.

Recognizing this urgency, the Society on Sarcopenia, Cachexia, and Wasting Disorders (SCWD) conducted a survey in 2022 among healthcare professionals, including physicians and dietitians, who specialize in caring for older adults with AA.^12^ Although many respondents demonstrated familiarity with the concept, the use of evidence‐based assessment tools for screening and diagnosing AA remains limited. Instead, appetite assessments often rely on subjective interviews and clinical judgement. In addition, a significant proportion of the respondents perceived appetite loss in older adults as inevitable and cited the lack of robust evidence for effective treatments as a barrier to intervention.

As new knowledge and expert opinions are updated on these issues, it is essential to have opportunities for active information gathering and education to better respond to these issues. Among the survey respondents, approximately 60% reported participation in voluntary nutrition education, and the remaining 40% did not have such opportunities. We raise a question regarding the potential influence of educational initiatives in addressing AA. However, detailed analyses of this influence are lacking.^12^

Therefore, this study aimed to analyse data from the aforementioned SCWD questionnaire survey of healthcare professionals caring for older adults in Japan, specifically examining the association between current understanding and management of AA and continuing education opportunity availability.

## Methods

The project was spearheaded by the SCWD and was conducted in collaboration with relevant societies in Europe, Japan, Latin America, and the United States.^12^ This international effort included several components: (1) a comprehensive literature review of both seminal and recent articles elucidating the definition, aetiology, and therapeutic strategies related to AA; (2) focus group discussions with clinical authorities; and (3) a survey of practicing healthcare professionals (HCPs) affiliated with the collaborating societies to obtain quantitative evidence. A mixed‐methods approach was used to ensure a comprehensive exploration. An International Advisory Board (IAB) was convened to review the current standards of care for anorexia in older adults and to identify deficiencies in professional practice among HCPs in their respective countries.

The formulation of the Japanese iteration of the questionnaire was overseen by the SCWD in collaboration with members of the Japan Geriatrics Society and the Japanese Association on Sarcopenia and Frailty. A web‐based survey was administered to relevant societies, including the Japan Geriatrics Society, the Japanese Association on Sarcopenia and Frailty, the Japanese Association of Rehabilitation Nutrition, the Japanese Society of Geriatric Pharmacy, the Japan Geriatric Therapy Society, the Japan Academy of Gerontological Nursing, the Japanese Society of Clinical Nutrition, and the Japan Home Nutrition Management Society.

### Survey

The quantitative survey comprised 26 items, including multiple‐choice, Likert scale, and open‐ended formats. The online questionnaire took approximately 20 min to complete and included various domains, including respondent demographics (four items), screening (five items), defining/diagnosing (five items), treating (four items), referrals (two items), and attitudes/perceptions (six items). The Japanese version of the survey was distributed electronically using QuestionPro between 16 June and 5 October 2022. Upon completion of the survey, data were extracted from QuestionPro© and analysed. To ensure confidentiality, all data were collected anonymously, and IP addresses were removed.

The selection criteria for respondents in this analysis were those who selected ‘Japan’ in response to item 4 ‘Please enter the country where you are currently working.’ Those who did not respond to the following items were excluded from the analysis: item 1, ‘Please indicate your current health care profession.’; item 10, ‘In the absence of an explicit cause such as acute illness, anorexia in older adults is most accurately defined as:’; item 15, ‘I use tools and resources such as evidence‐based guidelines developed by experts to care for my older patients with anorexia.’, item 26, ‘I participate in continuing education/continuing professional development on nutrition for (select all that apply).’

### Statistical analysis

Descriptive statistics, including means, standard deviations, and percentages, were included in the analysis, along with summaries of open‐ended responses. The response rates were assessed by dividing the single‐response items by the total number of respondents per item. For multiple response items (select all that apply), the response rate was calculated based on the number of respondents included in the analysis, regardless of any reduction in the number of respondents over time. For item 26, ‘I participate in continuing education/continuing professional development on nutrition for (select all that apply):’ respondents who selected ‘All patients’, ‘Older patients’, or ‘Older patients with anorexia’ were categorized as the ‘education group’, while those who selected ‘I do not engage in continuing education/continuing professional development on nutrition’ were categorized as the ‘non‐education group’. Single‐response items were compared between the two groups using the chi‐square test. For certain items, only physicians or registered dietitians, who typically have greater opportunities to intervene independently in patients experiencing AA, were selected for comparison based on their educational status. All reported *P*‐values were two‐tailed, and a significance level of <0.05 was considered statistically significant. All statistical analyses were performed using EZR (Saitama Medical Center, Jichi Medical University, Saitama, Japan),^13^ a modified version of R commander designed to include commonly used statistical functions in biostatistics based on R (The R Foundation for Statistical Computing, Vienna, Austria).

## Results

**Figure 1:**
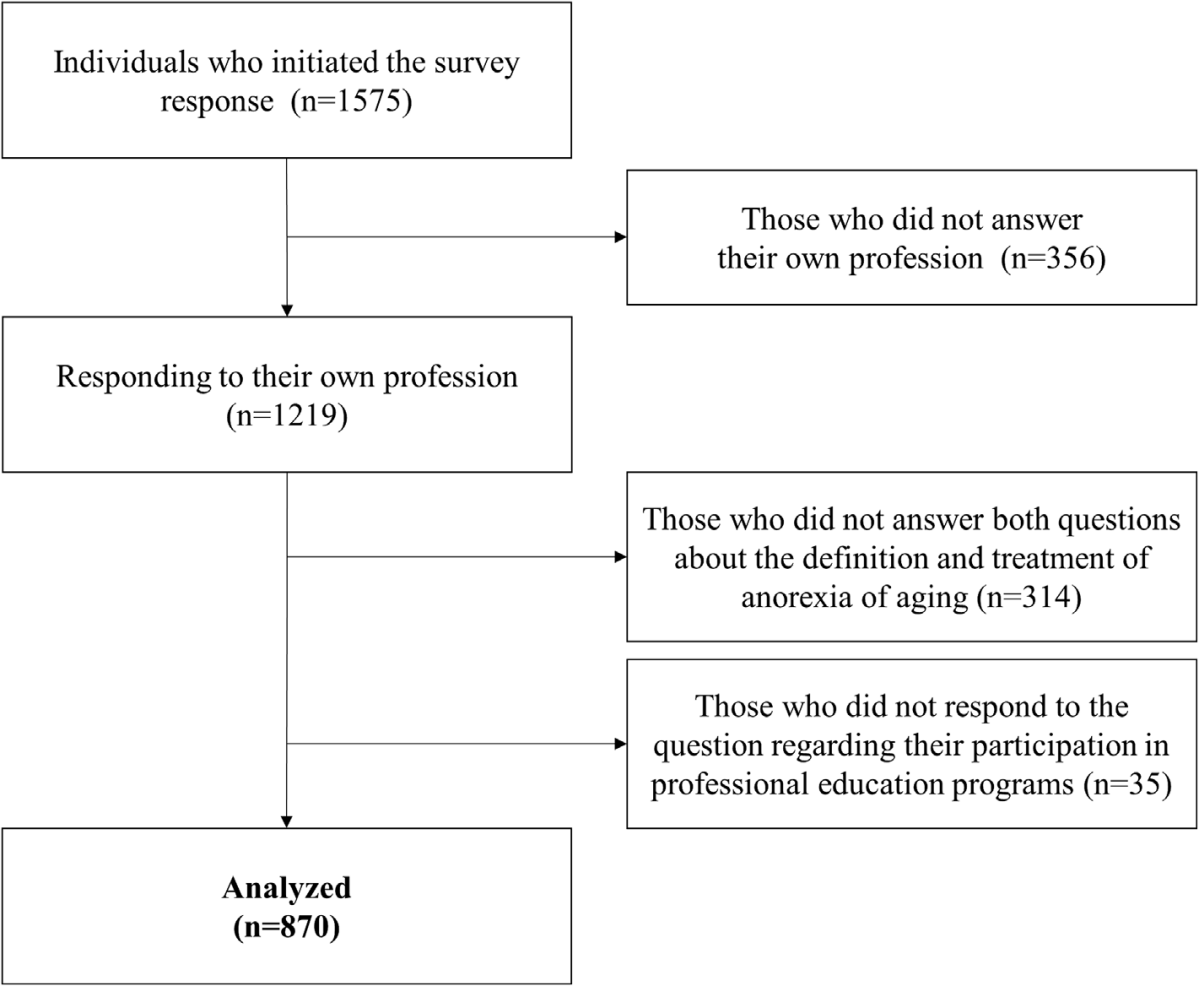
Flow chart of the selection of subjects in the Japanese subgroup analysis.

From the 1575 respondents initially surveyed, individuals who did not respond to questions about their profession (*n* = 356), definition and treatment of AA (*n* = 314), and participation in professional education programmes (*n* = 35) were excluded. A total of 870 respondents were included (Figure 1). The participant pool comprised 416 physicians (47.8%), 140 registered dietitians (16.1%), 120 rehabilitation therapists (physical therapists, occupational therapists, and speech‐language pathologists) (13.8%), 100 pharmacists (11.5%), 50 nurses (5.7%), and 44 other professions (5.1%). The most common specialties among the respondents were physical medicine/rehabilitation (16.4%) (19 physicians, 2.2% of the total), geriatrics (15.6%), and internal medicine (9.5%) (Table 1). Regarding the practice setting, most respondents (59.7%) worked primarily in hospitals, followed by home health care or clinics (18.4%) and nursing homes (11.3%) (data not shown). Of the respondents, 395 (45.4%) had participated in continuing education or professional development programmes focused on patient nutrition. The distribution of educational participants in each job category was as follows: 42.0% physicians, 24.3% registered dietitians, 14.4% therapists, 9.1% pharmacists, 5.1% nurses, and 5.1% other professions (data not shown).

**Table 1:** Respondent demographics

	*N*	%
Health profession		
Primary care or general practice physician/medical doctor	186	21.4%
Specialist physician/medical doctor	230	26.4%
Physician assistant	2	0.2%
Advanced practice nurse (e.g., nurse practitioner)	16	1.8%
Registered nurse/nurse	34	3.9%
Pharmacist	100	11.5%
Occupational therapist	14	1.6%
Physical therapist	76	8.7%
Dietitian/registered dietitian	140	16.1%
Social worker	2	0.2%
Psychologist	2	0.2%
Speech/language therapist	30	3.4%
Other mental health provider/counsellor	1	0.1%
Education specialist	4	0.5%
Other (free text)	33	3.8%
Specialty (if applicable)		
Allergy and immunology	3	0.3%
Cardiology	52	6.0%
Endocrinology	36	4.1%
Geriatrics	136	15.6%
Gastroenterology	15	1.7%
Internal medicine	83	9.5%
Neurology	55	6.3%
Oncology	2	0.2%
Otorhinolaryngology	4	0.5%
Physical medicine/rehabilitation	143	16.4%
Psychiatry	16	1.8%
Pulmonology	16	1.8%
Rheumatology	3	0.3%
General surgery	14	1.6%
Surgical specialty	15	1.7%
Other	169	19.4%
Missing system	108	12.4%
Primary practice location		
Academic medical centre	124	14.3%
Private hospital	272	31.3%
Public hospital	123	14.1%
Multispecialty group practice	14	1.6%
Long‐term care/nursing home	98	11.3%
Solo practice	57	6.6%
Specialty group practice	5	0.6%
Community‐based health centre/clinic	19	2.2%
Home health care	79	9.1%
Veterans administration medical centre/military facility	0	0.0%
National health service/government	11	1.3%
Other	49	5.6%
Missing system	19	2.2%

Table 2 presents the findings regarding AA screening. Significantly higher percentages of respondents in the education group reported assessing appetite and taking weight at each visit than those without training. The most commonly used screening tool was the Mini‐Nutritional Assessment Short Form (MNA‐SF), used by 49.1% of the respondents in the education group and 27.4% in the non‐education group. Conversely, almost half of the respondents in the non‐educated group and approximately one‐third in the educated group did not use any screening tools.

**Table 2:** Domain (screening): Screening for anorexia of aging

	Overall (*n* = 870)		Education group (*n* = 395)		Non‐education group (*n* = 475)		*P* value
	*N*	%	*N*	%	*N*	%	
Is appetite in older adults assessed at each visit?							
Yes	619	71.1%	302	76.5%	317	66.7%	0.006
No	165	19.0%	67	17.0%	98	20.6%	
Unsure	27	3.1%	7	1.8%	20	4.2%	
Not applicable for my practice setting	53	6.1%	17	4.3%	36	7.6%	
Missing system	6	0.7%	2	0.5%	4	0.8%	
Are older adults weighed at each visit using a weighing scale?							
Yes	341	39.2%	186	47.1%	155	32.6%	<0.001
No	428	49.2%	172	43.5%	256	53.9%	
Unsure	20	2.3%	8	2.0%	12	2.5%	
Not applicable for my practice setting	71	8.2%	25	6.3%	46	9.7%	
Missing system	10	1.1%	4	1.0%	6	1.3%	
How often should older adults be screened for appetite loss (select all that apply)?							
At each appointment	395	45.4%	198	50.1%	197	41.5%	
At least annually	154	17.7%	74	18.7%	80	16.8%	
When the older adult has lost a determined percentage of body weight (example: >10% body weight in the last 3 months)	465	53.4%	205	51.9%	260	54.7%	
When the older adult or family member expresses concern	242	27.8%	106	26.8%	136	28.6%	
I do not know	15	1.7%	5	1.3%	10	2.1%	
I do not screen older adults for appetite	16	1.8%	3	0.8%	13	2.7%	
Who is responsible for screening older adults for appetite loss (choose all that apply)?							
The primary treating physician	518	59.5%	231	58.5%	287	60.4%	
The nurse (nurse or advanced practice nurse)	508	58.4%	228	57.7%	280	58.9%	
Physician assistant	33	3.8%	13	3.3%	20	4.2%	
The dietitian/registered dietitian/nutritionist (non‐physician)	436	50.1%	226	57.2%	210	44.2%	
Medical or nursing assistant	51	5.9%	28	7.1%	23	4.8%	
Physical therapist	105	12.1%	52	13.2%	53	11.2%	
Pharmacist	73	8.4%	34	8.6%	39	8.2%	
Social worker	34	3.9%	18	4.6%	16	3.4%	
I do not know	24	2.8%	11	2.8%	13	2.7%	
No one	21	2.4%	4	1.0%	17	3.6%	
Other (free text)	76	8.7%	41	10.4%	35	7.4%	
Not applicable for my practice setting	10	1.1%	2	0.5%	8	1.7%	
The tools used in my practice setting to screen older adults for appetite loss include (select all that apply)							
Appetite, Hunger and Sensory Perception Questionnaire (AHSPQ)	10	1.1%	6	1.5%	4	0.8%	
Council on Nutrition Appetite Questionnaire (CNAQ)	23	2.6%	15	3.8%	8	1.7%	
Functional Assessment of Anorexia and Cachexia Therapy (FAACT)	6	0.7%	3	0.8%	3	0.6%	
Malnutrition Screening Tool (MST)	17	2.0%	9	2.3%	8	1.7%	
Malnutrition Universal Screening Tool (MUST)	33	3.8%	22	5.6%	11	2.3%	
Mini‐Nutritional Assessment Short Form (MNA‐SF)	324	37.2%	194	49.1%	130	27.4%	
Nutritional Risk Screening (NRS)	21	2.4%	11	2.8%	10	2.1%	
Rapid Geriatric Assessment (RGA)	5	0.6%	3	0.8%	2	0.4%	
Simplified Nutritional Appetite Questionnaire (SNAQ)	17	2.0%	11	2.8%	6	1.3%	
Visual Analogue Scale (VAS)	11	1.3%	3	0.8%	8	1.7%	
Informal clinical interview	56	6.4%	31	7.8%	25	5.3%	
Tool developed by my organization or association	19	2.2%	10	2.5%	9	1.9%	
We do not use a tool to screen older patients for appetite loss	350	40.2%	129	32.7%	221	46.5%	
I do not know	74	8.5%	20	5.1%	54	11.4%	
I do not screen older adults for appetite loss	49	5.6%	13	3.3%	36	7.6%	
Other (free text)	42	4.8%	21	5.3%	21	4.4%	

Data are presented as *N*, %, chi‐square test.

Table 3 presents items related to the definition and treatment of AA. Among the respondents, 52.5% defined AA as a loss of appetite and/or low food intake in older adults’. Regarding the use of evidence‐based tools and resources to treat AA, those with educational opportunities were more likely to use such tools and were less likely to report being unaware of them. In addition, a significantly higher proportion of respondents in the education group expressed confidence in the provision of nutritional and physical activity recommendations to older adults with anorexia. The most commonly selected general evidence‐based or consensus‐developed interventions for anorexia were the treatment of swallowing disorders (84.9%), addressing dentition issues (84.8%), and incorporating energy‐ and protein‐fortified foods into the diet (81.4%). In addition, more respondents in the education group reported having access to specialists for further assessment and treatment than those in the non‐education group.

**Table 3:** Domain (defining and treating): Defining and treating anorexia of aging

	Overall (*n* = 870)		Education group (*n* = 395)		Non‐education group (*n* = 475)		*P* value
	*N*	%	*N*	%	*N*	%	
In the absence of an explicit cause such as acute illness, anorexia in older adults is most accurately defined as							
Loss of appetite and/or low food intake in older adults	457	52.5%	207	52.4%	250	52.6%	0.830
Unintended weight loss in older adults	155	17.8%	71	18.0%	84	17.7%	
Sarcopenia or loss of muscle mass, strength and/or function	77	8.9%	38	9.6%	39	8.2%	
Nutrition risk, malnutrition or undernutrition in older adults	115	13.2%	53	13.4%	62	13.1%	
Frailty in geriatric patients	66	7.6%	26	6.6%	40	8.4%	
I use tools and resources such as evidence‐based guidelines developed by experts to care for my older patients with anorexia.							
Yes, all of the time	50	5.7%	37	9.4%	13	2.7%	<0.001
Yes, most of the time	160	18.4%	114	28.9%	46	9.7%	
Rarely	189	21.7%	100	25.3%	89	18.7%	
No, I prefer to use my own clinical judgement	259	29.8%	92	23.3%	167	35.2%	
No, I am not aware of tools and resources to care for my geriatric patients with anorexia	142	16.3%	35	8.9%	107	22.5%	
No, I do not use tools and resources because I do not have access to them	19	2.2%	5	1.3%	14	2.9%	
Not applicable for my professional role/responsibility	51	5.9%	12	3.0%	39	8.2%	
When a diagnosis of anorexia in older adults is made, evidence‐based or consensus developed interventions may include (select all that apply)							
Incorporating energy‐ and protein‐fortified foods in the diet	708	81.4%	334	84.6%	374	78.7%	
Recommending oral nutritional supplements (e.g., Boost and Ensure)	691	79.4%	312	79.0%	379	79.8%	
Addressing dentition issues	738	84.8%	338	85.6%	400	84.2%	
Treating swallowing disorders (if present)	739	84.9%	343	86.8%	396	83.4%	
Prescribing appetite stimulants (e.g., megace and dronabinol)	166	19.1%	85	21.5%	81	17.1%	
Prescribing antidepressants	263	30.2%	134	33.9%	129	27.2%	
Prescribing physical exercise	434	49.9%	197	49.9%	237	49.9%	
Prescribing nutritional counselling	508	58.4%	238	60.3%	270	56.8%	
Revising current prescriptions that are causing side effects	614	70.6%	282	71.4%	332	69.9%	
Treating constipation	609	70.0%	278	70.4%	331	69.7%	
Reviewing already prescribed medications	616	70.8%	281	71.1%	335	70.5%	
Referring to specialist for psychosocial support	298	34.3%	136	34.4%	162	34.1%	
Referring to support services (e.g., social worker, financial counsellor, and transportation assistance)	355	40.8%	155	39.2%	200	42.1%	
Screening for abuse and/or neglect	247	28.4%	111	28.1%	136	28.6%	
Other (free text)	3	0.3%	0	0.0%	3	0.6%	
I do not know	6	0.7%	2	0.5%	4	0.8%	
Not applicable for my professional role/responsibility	10	1.1%	3	0.8%	7	1.5%	
I am confident in providing nutrition recommendations for older patients with anorexia.							
Strongly agree	199	22.9%	116	29.4%	83	17.5%	<0.001
Agree	395	45.4%	198	50.1%	197	41.5%	
Neither agree nor disagree	193	22.2%	62	15.7%	131	27.6%	
Disagree	45	5.2%	13	3.3%	32	6.7%	
Strongly disagree	12	1.4%	1	0.3%	11	2.3%	
Not applicable for my professional role/responsibility	22	2.5%	4	1.0%	18	3.8%	
Missing system	4	0.5%	1	0.3%	3	0.6%	
I am confident in providing physical activity recommendations for older patients with anorexia.							
Strongly agree	151	17.4%	88	22.3%	63	13.3%	<0.001
Agree	413	47.5%	210	53.2%	203	42.7%	
Neither agree nor disagree	212	24.4%	73	18.5%	139	29.3%	
Disagree	55	6.3%	15	3.8%	40	8.4%	
Strongly disagree	6	0.7%	1	0.3%	5	1.1%	
Not applicable for my professional role/responsibility	26	3.0%	5	1.3%	21	4.4%	
Missing system	7	0.8%	3	0.8%	4	0.8%	
There are sufficient specialists available for me to refer my older adult patients with anorexia for additional assessment and/or treatment.							
Yes, all of the time	63	7.2%	42	10.6%	21	4.4%	<0.001
Yes, most of the time	287	33.0%	153	38.7%	134	28.2%	
Rarely	166	19.1%	83	21.0%	83	17.5%	
No	267	30.7%	87	22.0%	180	37.9%	
Not applicable for my professional role/responsibility	83	9.5%	26	6.6%	57	12.0%	
Missing system	4	0.5%	4	1.0%	0	0.0%	

Data are presented as *N*, %, chi‐square test.

Table 4 presents items related to attitudes and perceptions about AA. Just over 50% of respondents agreed with the statements ‘Anorexia is unavoidable in geriatric patients’ and ‘Lack of high‐quality evidence to guide the care and treatment of older patients with anorexia makes it challenging for me as a clinician to choose treatment’. In addition, 46.6% of those with educational opportunities reported having access to a team of professionals experienced in caring for older adults with anorexia, compared with 24.2% of those without educational opportunities.

**Table 4:** Domain (attitudes and perceptions): Perceptions and attitudes in the care of older patients with anorexia of aging

	Overall (*n* = 870)		Education group (*n* = 395)		Non‐education group (*n* = 475)		*P* value
	*N*	%	*N*	%	*N*	%	
Anorexia is unavoidable in geriatric patients.							
Strongly agree	146	16.8%	76	19.2%	70	14.7%	0.307
Agree	363	41.7%	164	41.5%	199	41.9%	
Neither agree nor disagree	235	27.0%	101	25.6%	134	28.2%	
Disagree	119	13.7%	50	12.7%	69	14.5%	
Strongly disagree	6	0.7%	4	1.0%	2	0.4%	
Missing system	1	0.1%	0	0.0%	1	0.2%	
The regular use of standardized tools to evaluate older patients for weight loss is critical.							
Strongly agree	355	40.8%	182	46.1%	173	36.4%	0.012
Agree	442	50.8%	191	48.4%	251	52.8%	
Neither agree nor disagree	57	6.6%	17	4.3%	40	8.4%	
Disagree	11	1.3%	3	0.8%	8	1.7%	
Strongly disagree	2	0.2%	1	0.3%	1	0.2%	
Missing system	3	0.3%	1	0.3%	2	0.4%	
Lack of high‐quality evidence to guide the care and treatment of older patients with anorexia makes it challenging for me as a clinician to choose treatment.							
Strongly agree	94	10.8%	41	10.4%	53	11.2%	0.046
Agree	368	42.3%	174	44.1%	194	40.8%	
Neither agree nor disagree	236	27.1%	91	23.0%	145	30.5%	
Disagree	142	16.3%	71	18.0%	71	14.9%	
Strongly disagree	27	3.1%	17	4.3%	10	2.1%	
Missing system	3	0.3%	1	0.3%	2	0.4%	
I have access to an interprofessional team with experience in the care of older adults with anorexia.							
Yes, all of the time	66	7.6%	45	11.4%	21	4.4%	<0.001
Yes, most of the time	233	26.8%	139	35.2%	94	19.8%	
Rarely	192	22.1%	94	23.8%	98	20.6%	
No	343	39.4%	109	27.6%	234	49.3%	
Not applicable for my professional role/responsibility	33	3.8%	6	1.5%	27	5.7%	
Missing system	3	0.3%	2	0.5%	1	0.2%	
I involve caregivers such as family members as collaborators in supporting the older adult with anorexia.							
Strongly agree	213	24.5%	112	28.4%	101	21.3%	<0.001
Agree	539	62.0%	250	63.3%	289	60.8%	
Neither agree nor disagree	100	11.5%	29	7.3%	71	14.9%	
Disagree	12	1.4%	2	0.5%	10	2.1%	
Strongly disagree	3	0.3%	1	0.3%	2	0.4%	
Missing system	3	0.3%	1	0.3%	2	0.4%	

Data are presented as *N*, %, chi‐square test.

Tables 5, 6, 7 present the results for physicians and registered dietitians, focusing mainly on items where differences were observed due to participation in the educational programme. For screening for anorexia in older adults, a higher percentage of physicians refrained from using screening tools compared to the total respondent pool, with 40.4% in the education group and 59.6% in the non‐education group. In contrast, more than half of the registered dietitians in both groups used the MNA‐SF (Table 5). Regarding the treatment of anorexia in older patients, the majority of physicians with no education reported relying on their own clinical judgement (Table 6). In addition, consultation with specialists and teams was significantly higher among both physicians and registered dietitians with education, with particularly pronounced differences among physicians based on education status (Table 7).

**Table 5:** Comparison of screening for anorexia of aging in physicians and registered dietitians with and without education

	Physicians (*n* = 416)					Registered dietitians (*n* = 140)				
	Education group (*n* = 166)		Non‐education group (*n* = 250)		*P* value	Education group (*n* = 96)		Non‐education group (*n* = 44)		*P* value
	*N*	%	*N*	%		*N*	%	*N*	%	
Is appetite in older adults assessed at each visit?										
Yes	134	80.7%	197	78.8%	0.250	79	82.3%	30	68.2%	0.074
No	32	19.3%	45	18.0%		8	8.3%	8	18.2%	
Unsure	0	0.0%	2	0.8%		1	1.0%	3	6.8%	
Not applicable for my practice setting	0	0.0%	4	1.6%		8	8.3%	3	6.8%	
Missing system	0	0.0%	2	0.8%		0	0.0%	0	0.0%	
Are older adults weighed at each visit using a weighing scale?										
Yes	88	53.0%	100	40.0%	0.026	55	57.3%	19	43.2%	0.268
No	77	46.4%	143	57.2%		26	27.1%	19	43.2%	
Unsure	0	0.0%	1	0.4%		4	4.2%	1	2.3%	
Not applicable for my practice setting	0	0.0%	4	1.6%		10	10.4%	4	9.1%	
Missing system	1	0.6%	2	0.8%		1	1.0%	1	2.3%	
The tools used in my practice setting to screen older adults for appetite loss include (select all that apply)										
Appetite, Hunger and Sensory Perception Questionnaire (AHSPQ)	6	3.6%	3	1.2%		0	0.0%	0	0.0%	
Council on Nutrition Appetite Questionnaire (CNAQ)	7	4.2%	2	0.8%		6	6.3%	3	6.8%	
Functional Assessment of Anorexia and Cachexia Therapy (FAACT)	3	1.8%	2	0.8%		0	0.0%	0	0.0%	
Malnutrition Screening Tool (MST)	7	4.2%	5	2.0%		1	1.0%	0	0.0%	
Malnutrition Universal Screening Tool (MUST)	7	4.2%	5	2.0%		12	12.5%	1	2.3%	
Mini‐Nutritional Assessment Short Form (MNA‐SF)	69	41.6%	48	19.2%		59	61.5%	22	50.0%	
Nutritional Risk Screening (NRS)	2	1.2%	7	2.8%		4	4.2%	1	2.3%	
Rapid Geriatric Assessment (RGA)	1	0.6%	1	0.4%		0	0.0%	0	0.0%	
Simplified Nutritional Appetite Questionnaire (SNAQ)	6	3.6%	2	0.8%		5	5.2%	1	2.3%	
Visual Analogue Scale (VAS)	0	0.0%	5	2.0%		2	2.1%	0	0.0%	
Informal clinical interview	17	10.2%	17	6.8%		5	5.2%	1	2.3%	
Tool developed by my organization or association	5	3.0%	5	2.0%		4	4.2%	4	9.1%	
We do not use a tool to screen older patients for appetite loss	67	40.4%	149	59.6%		22	22.9%	12	27.3%	
I do not know	2	1.2%	12	4.8%		4	4.2%	3	6.8%	
I do not screen older adults for appetite loss	5	3.0%	17	6.8%		3	3.1%	3	6.8%	
Other (free text)	5	3.0%	13	5.2%		8	8.3%	2	4.5%	

Data are presented as *N*, %, chi‐square test.

**Table 6:** Comparison of treating for anorexia of aging in physicians and registered dietitians with and without education

	Physicians (*n* = 416)					Registered dietitians (*n* = 140)				
	Education group (*n* = 166)		Non‐education group (*n* = 250)		*P* value	Education group (*n* = 96)		Non‐education group (*n* = 44)		*P* value
	*N*	%	*N*	%		*N*	%	*N*	%	
I use tools and resources such as evidence‐based guidelines developed by experts to care for my older patients with anorexia.										
Yes, all of the time	10	6.0%	8	3.2%	<0.001	15	15.6%	2	4.5%	0.140
Yes, most of the time	48	28.9%	16	6.4%		35	36.5%	11	25.0%	
Rarely	42	25.3%	39	15.6%		23	24.0%	12	27.3%	
No, I prefer to use my own clinical judgement	50	30.1%	129	51.6%		13	13.5%	10	22.7%	
No, I am not aware of tools and resources to care for my geriatric patients with anorexia	13	7.8%	47	18.8%		6	6.3%	6	13.6%	
No, I do not use tools and resources because I do not have access to them	2	1.2%	6	2.4%		1	1.0%	2	4.5%	
Not applicable for my professional role/responsibility	1	0.6%	5	2.0%		3	3.1%	1	2.3%	
I am confident in providing nutrition recommendations for older patients with anorexia.										
Strongly agree	52	31.3%	39	15.6%	<0.001	37	38.5%	19	43.2%	0.201
Agree	81	48.8%	108	43.2%		50	52.1%	16	36.4%	
Neither agree nor disagree	24	14.5%	75	30.0%		8	8.3%	8	18.2%	
Disagree	6	3.6%	23	9.2%		1	1.0%	1	2.3%	
Strongly disagree	1	0.6%	2	0.8%		0	0.0%	0	0.0%	
Not applicable for my professional role/responsibility	1	0.6%	1	0.4%		0	0.0%	0	0.0%	
Missing system	1	0.6%	2	0.8%		0	0.0%	0	0.0%	
I am confident in providing physical activity recommendations for older patients with anorexia.										
Strongly agree	41	24.7%	35	14.0%	<0.001	16	16.7%	7	15.9%	0.043
Agree	89	53.6%	109	43.6%		58	60.4%	17	38.6%	
Neither agree nor disagree	30	18.1%	78	31.2%		15	15.6%	17	38.6%	
Disagree	5	3.0%	20	8.0%		2	2.1%	2	4.5%	
Strongly disagree	0	0.0%	2	0.8%		1	1.0%	1	2.3%	
Not applicable for my professional role/responsibility	0	0.0%	2	0.8%		2	2.1%	0	0.0%	
Missing system	1	0.6%	4	1.6%		2	2.1%	0	0.0%	

Data are presented as *N*, %, chi‐square test.

**Table 7:** Comparison of referrals for anorexia of aging in physicians and registered dietitians with and without education

	Physicians (*n* = 416)					Registered dietitians (*n* = 140)				
	Education group (*n* = 166)		Non‐education group (*n* = 250)		*P* value	Education group (*n* = 96)		Non‐education group (*n* = 44)		*P* value
	*N*	%	*N*	%		*N*	%	*N*	%	
There are sufficient specialists available for me to refer my older adult patients with anorexia for additional assessment and/or treatment.										
Yes, all of the time	18	10.8%	13	5.2%	<0.001	13	7.8%	0	0.0%	0.029
Yes, most of the time	66	39.8%	73	29.2%		38	22.9%	18	7.2%	
Rarely	39	23.5%	46	18.4%		20	12.0%	6	2.4%	
No	33	19.9%	102	40.8%		15	9.0%	13	5.2%	
Not applicable for my professional role/responsibility	8	4.8%	16	6.4%		9	5.4%	7	2.8%	
Missing system	2	1.2%	0	0.0%		1	0.6%	0	0.0%	
I have access to an interprofessional team with experience in the care of older adults with anorexia.										
Yes, all of the time	19	11.4%	11	4.4%	<0.001	12	7.2%	3	1.2%	0.034
Yes, most of the time	58	34.9%	50	20.0%		32	19.3%	10	4.0%	
Rarely	46	27.7%	61	24.4%		23	13.9%	8	3.2%	
No	42	25.3%	122	48.8%		25	15.1%	23	9.2%	
Not applicable for my professional role/responsibility	1	0.6%	6	2.4%		4	2.4%	0	0.0%	
Missing system	0	0.0%	0	0.0%		0	0.0%	0	0.0%	

Data are presented as *N*, %, chi‐square test.

## Discussion

In this study, a Japanese sub‐analysis of global surveys was conducted among healthcare professionals involved in anorexia treatment in older adults.^12^ Our findings shed light on several key aspects of AA. Despite the high frequency of appetite assessment, over 40% of the respondents did not use professionally developed tools for screening and treatment. In addition, more than half of the respondents believed that appetite loss is inevitable in older patients and lamented the lack of high‐quality evidence for treatment, which poses a challenge in initiating interventions for AA. In addition, a significant proportion of the respondents did not participate in continuing education or professional development programmes related to nutrition. Notably, participation in such educational initiatives correlated with more standardized measures of response to screening and treatment for AA, as well as improved access to specialist referrals and interdisciplinary teams. These findings underscore the importance of continuous education in this field.

In this study, there were notable disparities in the screening and treatment of appetite loss among older adults as a function of participation in continuing education and professional development programmes focused on nutrition. In our Japanese survey, less than half the respondents participated in continuing education opportunities related to nutrition, a proportion lower than that observed in the overall survey dataset.^12^ This may be due to the lack of an established continuing medical education system in Japan compared to other countries, which may have resulted in fewer opportunities for Japanese respondents to participate in educational efforts. Individuals with educational opportunities demonstrated higher frequencies of appetite assessment and weighing as well as increased use of evidence‐based screening tools. They also demonstrated greater confidence in recommending nutritional and physical activity interventions for older adults with anorexia during treatment. Additionally, respondents with educational opportunities were more likely to report access to a team of professionals experienced in managing anorexia among older adults. Those with educational opportunities likely recognized the importance of a collaborative team approach for treating anorexia in older patients. In addition, the presence of experts in their environments may have acted as a catalyst for their participation in educational programmes. Thus, the interplay between environmental factors and educational opportunities may have a notable influence on the quality of the evaluation and treatment of AA.

Next, results highlight the need for a comprehensive review of clinical nutrition education programmes in Japan. Although the MNA‐SF has emerged as the most widely used screening tool among individuals with educational opportunities, it primarily assesses nutritional status, with decreased food intake being the only assessment parameter.^14^ The use of appetite‐specific tools such as the Functional Assessment of Anorexia and Cachexia Therapy (FAACT),^15^, ^16^ Council on Nutrition Appetite Questionnaire (CNAQ),^17^, ^18^ and Simplified Nutritional Appetite Questionnaire (SNAQ)^17^, ^19^ remained minimal among those with educational opportunities as well as among registered dietitians. In addition, even among those who received continuing education, approximately 30% did not incorporate evidence‐based tools or materials into the screening and treatment protocols for anorexia in older adults. More than half of the respondents acknowledged the paucity of high‐quality evidence on AA, indicating the critical need for further education in this area. While existing nutrition education programmes in Japan, such as TNT‐Geriatric^20^ and TNT‐Rehabilitation,^21^ and educational programmes of clinical nutrition‐related societies provide adequate content on nutritional status assessment and disease‐specific nutrition management, they offer relatively limited coverage on the assessment and treatment of anorexia. Because AA is due to a variety of factors, including age‐related changes in the gastrointestinal tract and sensory organs, as well as the influence of diseases and medications,^8^ medical interventions for anorexia must adopt a comprehensive approach based on analytical, diagnostic reasoning, rather than simply attributing it to aging or relying solely on oral nutritional supplementation.^22^ There is an urgent need to revise existing training programmes to incorporate the FAACT, CNAQ, SNAQ, and other tools specific to anorexia and appetite assessment. In addition, the curricula should be revised to equip health professionals with diagnostic reasoning‐based methodologies. Furthermore, educational programmes in geriatrics have been shown to benefit from multidisciplinary and interdisciplinary content, the incorporation of group learning, and the use of case studies.^23^ Notably, evidence from a one‐day educational programme on frailty among primary care providers demonstrated sustained improvements in participants' perceptions of frailty and screening practices.^24^ However, there is a lack of research evaluating the effectiveness of educational programmes focused on AA, which warrants further investigation.

The significance of this study lies in its use of a web‐based survey format, which facilitated responses not only from hospital staff but also from a wide range of healthcare professionals involved in caring for older patients in various settings, including nursing homes and home care. By juxtaposing knowledge and practices regarding AA with the availability of educational opportunities in nutrition management for patients, this study identified pertinent issues related to educational programmes in this area.

However, this study has several limitations. First, the survey respondents were primarily members of academic societies related to geriatrics, most of whom were healthcare professionals with some familiarity with the management of AA. Therefore, the results may not be fully representative of clinical practice. In addition, the response rate for the survey was moderate, with only 56.7% (893/1575) of all respondents providing complete responses without omissions, suggesting a potential bias in the dataset.

## Conclusions

Healthcare professionals' participation in continuing education programmes on clinical nutrition is associated with their responsiveness in screening, diagnosing, and treating older adults with anorexia and the availability of expert teams, which may affect the quality of care for AA. Nutrition education may support the confidence of health care professionals working with older adults in AA with complex clinical signs and encourage them to conduct evidence‐based assessments.

## Conflict of interest

MA received research funding from Eisai, Kracie Pharma, Mitsubishi‐Tanabe Pharma, and Tsumura and lecture fees from Bayer HealthCare, Daiichi Sankyo, Toa Eiyo, and Towa Pharmaceutical. IA has received consultancy fees from Pfizer. AC has received honoraria and/or lecture fees from AstraZeneca, Boehringer Ingelheim, Menarini, Novartis, Servier, Vifor, Abbott, Actimed, Arena, Cardiac Dimensions, Corvia, CVRx, Enopace, ESN Cleer, Faraday, Impulse Dynamics, Respicardia, and Viatris. SDA has received grants and personal fees from Vifor and Abbott Vascular and personal fees for consultancies, trial committee work and/or lectures from Actimed, Amgen, AstraZeneca, Bayer, Boehringer Ingelheim, BioVentrix, Brahms, Cardiac Dimensions, Cardior, Cordio, CVRx, Cytokinetics, Edwards, Farraday Pharmaceuticals, GSK, HeartKinetics, Impulse Dynamics, Novartis, Occlutech, Pfizer, Repairon, Sensible Medical, Servier, Vectorious, and V‐Wave. He has been named co‐inventor of two patent applications regarding MR‐proANP (DE 102007010834 and DE 102007022367), but he does not benefit personally from the related issued patents. The rest of the authors declare no conflict of interest.
